# Eyesight: Its Care during Infancy and Youth

**Published:** 1893-02

**Authors:** L. Webster Fox


### SELECTIONS.

### Eyesight: Its Care During Infancy And Youth.

A Lecture delivered before the Franklin Institute.

BY L. WEBSTER FOX, M.D.

Vision is the most useful of all the senses. It is the one gift which we should cherish and guard the most. And at no time in one's life is it more precious than in infancy and youth.

In infancy, when the child is developing, the one great avenue to the unfolding, or, more properly speaking, the development of the intellect is through the eye. The eye at this period holds in abeyance all the other senses. The child, when insensible to touch, taste, smell or hearing, will become aroused to action by a bright light or bright colors, or the movement of any illuminated object, proving to all that light is essential to the development of the first and most important sense. Again, the infant of but six days of age will recognize a candle flame, while its second sense and second in importance to its development—hearing—will not be recognized for *six* weeks to two months. Taste, touch, and smell follow in regular sequence. Inasmuch as light makes thus early an impression on the delicate organ of vision, how necessary it behooves us to guard the infant from too bright lights or too much exposure in our bright climate. Mothers—not only the young mother with her first child, but also those who have had several children—are too apt to try to quiet a restless child by placing it near a bright flame; much evil to the future use of those eyes is the outgrowth of such a pernicious habit. Light throws into action certain cells of that wonderful structure of the eye, the retina, and an over-stimulus perverts the action of those cells. The result is that by this over-stimulation the seeds of future trouble are sown. Let the adult gaze upon the arc of an electric light or into the sun, and for many moments, nay hours, that individual has dancing before his vision scintillations and phosphenes. His direct vision becomes blurred, and, as in the case of a certain individual I have in mind, there may be a permanent loss of sight. Parents should take the first precaution in the child's life, and not expose it to a light too bright or glaring. When in the open air let the child's eyes be protected from the direct rays of the sun. While it is impossible to give all children the advantage of green fields and out-door ramblings, yet nature never intended that civilization should debar the innocent child from such surroundings.

An anecdote is related of a French ophthalmic surgeon, that a distinguished patient applied to him for relief from a visual defect; the surgeon advised him to go into the country and look out upon the green fields; the green color with its soothing effect soon brought about a restoration of vision. What I wish to illustrate by this anecdote is that children should be allowed the green fields as their best friend in early life, it tones up the system and rests the eye. After out-door exercise and plenty of it, we should turn our attention to the home surroundings of our little ones. The overheated rooms of the average American home I am sure have more to do with the growing tendency of weak eyes than we feel like admitting. Look at these frail hot-house plants, and can any one believe that such bodies nourished in almost pestilential atmosphere can nourish such delicate organs of vision, and keep them ready for the enormous amount of work each little eye performs daily? The brain developing so rapidly wills with an increasing rapidity the eye to do increasing duties. Note the result: a tendency to impoverished circulation first, and the eye with its power to give the brain a new picture in an infinitesimal short space of time means lightning-like circulation—the eye must give way by its own exhaustion.

Civilization is the progenitor of many eye diseases.

After a boy has grown to that age when it becomes necessary for him to begin the education prescribed by the wise men, obstacles are placed in his way to aid again in causing deterioration of vision. It is not so much the overcrowded condition of our school-rooms as the enormous amount of work that causes deterioration of sight. Our children begin their school-life at a time when they are too young. A child at six years of age who is forced to study all day or even a part of a day will not run the same race that one will who commences his studies at ten—all things being equal. The law prescribes that so much time must be devoted to study, so many forms must be passed, so many books must be read, so many pages of composition written—all probably in badly-lighted rooms, or by artificial light. Note the effect: first, possibly, distant vision gives way; the teacher, sympathizing with the overburdened child, tries to make the burden lighter by changing his position in the room or placing him under the cross-light from a window; as the evil progresses the child is taken to an ophtalmic surgeon and the inevitable result—glasses —rightly called "crutches for the eyes"—are given. What would be thought of a cause which would weaken the legs of that boy so that he would have to use crutches to carry him through life? If civilization be responsible for an evil, let our efforts be put forth in finding a remedy for that evil.

A discussion, in a recent number of the *British Medical Journal*,* on "The Claims and Limitations of Physical Education in Schools," has many valuable hints which should be followed by educators in this country. Dr. Carter, in the leading paper on this subject, makes the pregnant remark: "If the hope is entertained of building up a science of education, the medical profession must combine with the profession of teaching, in order to direct investigation and to collect material essential to generalization. Without such co-operation educational workers must continue to flounder in the morasses of empiricism, and be content to purchase relative safety at the cost of slow progress, or no progress at all." In other words, an advisory medical board should co-exist with our board of public education, to try to hold in check or prevent a further "cruelty in trying to be kind." Private institutions of educations recognize the importance of physical training and development, and in such institutions the deterioration of vision is in proportion, less than in institutions where physical training is not considered. In one school of over 200

*November 1st, 1890.

middle-class girls, Dr. Carter found that, during a period of six years, no fewer than 10 per cent. of the total number of girls admitted during that time have been compelled to take one or more terms' leave of absence, and of the present number 28 per cent. have medical certificates exempting them from gymnastic exercise, and 10.25 per cent. of the total present number wear eye-glasses of some kind or other. From my own experience the same number of students in our schools would show about the same percentage of visual defects. These questions are of such growing importance that not only instructors, but the medical fraternity should not rest until these evils are eradicated.

Dr. J. W. Ballantyne, of Edinburgh, in a lecture† on diseases of infancy and childhood, says: "The education of the young people of a nation is to that nation a subject of vital importance." The same writer quotes the startling statement made by Professor Pfluger, that of 45,000 children examined in Germany more than one-half were suffering from defective eyesight, while in some schools the proportion of the short-sighted was 70 or 80 per cent., and crowning all was the Heidelberg Gymnasium, with 100 per cent. These figures, the result of a careful examination, are simply startling, and almost make one feel that 'twere better to return to the old Greek method of teaching by word of mouth.

The abuse of tobacco leads to impairment of vision in the growing youth. Cigarette-smoking is an evil. I am inclined to believe that the poisons inhaled arrest the growth of boys; surely it prevents a mental development, and when carried to excess affects vision more by lessening the power of nerve conduction than acting directly on the eye.

It is not the one cigarette which the boy smokes that does the harm, but it is the one, two, or three packages smoked daily. This excessive smoking thoroughly perverts all the functions which should be at their best to aid this growing youth. First we have failing digestion, restless nights, suspension of growth, lack of mental development, the loss of nerve tone, loss of the power of accommodation in vision, failing sight, headaches, en-

* *Lancet*, November 1st, 1890.

feeblement of the heart. Let a man who is a habitual smoker of cigars attempt to smoke even one package of cigarettes and he will complain of nausea, dry throat, and loss of appetite. If a strong man is so much affected by this poison, how much less can a boy resist the inroads of such poisons. In Germany the law forbids the sale of cigarettes to growing boys. New York State has a similar law, and why should our own or any other State be behind in passing prohibitory laws against this evil; and this is a growing evil.

I have never seen a case of tobacco amblyopia in boyhood, but such a condition is not infrequent in adults. In boys the action of nicotine acts especially upon the heart, the impulse is rendered weaker and intermittent, and many young boys lay the seed of organic disease which sooner or later culminates fatally. Boys should be prohibited from smoking, first by their parents, second by law, but not such laws whose enforcement is a failure, third by placing a heavy fine upon dealers who sell to minors. The pernicious evil of intoxication is no less an evil upon the nervous system of a youth than is the habit of cigarette smoking, but fortunately this habit is less common. Having traced from aborginal man to the present civilized individual the cause of his myopia, what must we do to prevent a further deterioration of vision? Unfortunately, the physician of our country is not, as I am told, like the Japanese physician. Our medical men are called to attend people who are ill and to try to get them well—the Japanese physician is paid only to keep his patients in health.

The first effort parents should make is to see that their children have plenty of out-door exercise. Good warm clothing in winter and light-texture cloth in summer. A great difference of opinion exists as to the age at which a child should begin its studies. I feel sure that the boy who commences his studies at ten will far outrun the one who commences study at six. Every child should commence his lessons in the best kindergarten, the nursery. Let object lessons be his primer—let him be taught by word of mouth—then, when his brains is what it should be for a boy of ten, his eyes will be the better able to bear the fatigue of the burdens which will be forced upon him. Listen to what Milton has left on record as a warning to those young boys or girls who insist upon reading or studying at night with bad illumination:

"My father destined me, from a child, for the pursuits of polite learning, which I prosecuted with such eagerness that, after I was twelve years old, I rarely retired to bed, from my lucubrations, till midnight. This was the first thing which proved pernicious to my eyes, to the natural weakness of which were added frequent headaches." Milton went blind when comparatively a young man, and it was always to him a great grief. Galileo, the great astronomer, also went blind by overwork. It was written of him, "The noblest eye which ever nature made is darkened—an eye so privileged, and gifted with such rare powers, that it may truly be said to have seen more than the eyes of all that are gone, and to have opened the eyes of all that are to come."

In the wake of advanced education stalks the spectacle age. Any one watching a passing crowd cannot fail but note the great number of people wearing spectacles. Unfortunately it is not limited to adults, but our youths of both sexes go to make up this army of ametropes.

At what age should children first wear glasses? This is a much debatable question. Where there is simply a defect of vision I should never prescribe a pair of glasses for a child under ten years of age. A child under this age runs many risks of injury to the eyeball by accident to the glasses, and to cut the eye with glass is a very serious affair. Rather let a child go without study, or even with impaired vision, than run the risk of a permanent loss of sight.

Another source of evil I must call your attention to, and that is the indiscriminate use of glasses given by itinerant vendors of spectacles who claim a thorough knowledge of the eye, who make examination free, but charge double price for glasses.

Persons, before submitting themselves into the hands of opticians, should know that they are not suffering from any incipient disease of their eyes. I do not for a moment claim that a practical optician cannot give you a pair of glasses which will make you see—he does nothing more than hand you a number of pairs of glasses and you select the one pair which you think answers the purpose. How can any one but a medical man know that the impairment of vision does not arise from diminished sensibility of the retina? If so, the glasses just purchased, which may be comfortable for a time, may cause an irreparable loss of vision. Every ophthalmic surgeon will tell you that he has had a number of such cases. Do not be misguided by purchasing cheap spectacles. Glasses advertised as having "remarkable qualities" are always to be passed by. They have "remarkable qualities;" they always leave the person wearing them worse at the end of a few months. Whenever an eye finds relief in a shaded or colored glass, something is going wrong with the interior of that eye. Seek advice, but do not trust the eyes of yourself, much less those of your children, in the hands of the opticians who advertise their examinations free.

Such individuals should be brought before a tribunal, and the matter sifted as to whether the sense of sight is less to be taken care of than if that same patient were ill with pneumonia, and a druggist were to prescribe remedies which might or might not aid this patient. If one man must comply with the law, why should not the other? Our medical colleges are lengthening the course of studies; the advances in the various departments of science demand this. It is by the aid of the ophthalmoscope that many obscure diseases are diagnosed, and while it is impossible for every young man who obtains a diploma to become thoroughly proficient in the use of this instrument, yet the eye shows to him many conditions which guide him to the road of successful treatment. Think of a case of optic neuritis--inflammation of the optic nerve--going to an optician and fitting one set of glasses after another until the patient suddenly discovers that blindness is inevitable. Many individuals, and very intelligent ones at that, think that so long as a glass makes them see, that is all they need. When we know that scarely two eyes are alike, we can at once feel that it is very important that each eye should be properly adjusted for a glass; by this we are sure of having comfort in reading and preserving vision.

To aid in a feeble way for the protection of posterity I have formulated ten rules on the preservation of vision:

1. Do not allow light to fall upon the face of a sleeping infant.

2. Do not allow babies to gaze at a bright light.

3. Do not send children to school before the age of ten.

4. Do not allow children to keep their eyes too long on a near object, at any one time.

5. Do not allow them to study much by artificial light.

6. Do not allow them to use books with small type.

7. Do not allow them to read in a railway carriage.

8. Do not allow boys to smoke tobacco, especially cigarettes.

9. Do not necessarily ascribe headaches to indigestion, the eyes may be the exciting cause.

10. Do not allow the itinerant spectacle vendor to prescribe glasses.—*Journal of the Franklin Institue, September*, 1891.

